# Does the coexistence of literal and figurative meanings in metaphor comprehension yield novel meaning?: Empirical testing based on quantum cognition

**DOI:** 10.3389/fpsyg.2023.1146262

**Published:** 2023-03-30

**Authors:** Miho Fuyama

**Affiliations:** College of Letters, Ritsumeikan University, Kyoto, Japan

**Keywords:** compositionality, conceptual combination, metaphor, superposition, quantum cognition

## Abstract

Metaphor comprehension is a creative process that may lead to the emergence of novel meaning. Several studies have examined the emergence according to the interaction between the topic and vehicle. We focused on the other type of emergence in metaphor comprehension: the interaction between the literal and figurative meanings. This article hypothesized that the whole meaning of some metaphorical sentences can be regarded as a superposition state of their literal and figurative meanings, which cannot be reduced to the simple composition of each meaning. To test this hypothesis, we applied QQ equality to metaphor comprehension and conducted an experiment using 21 metaphorical sentences and 1,000 participants. The model comparisons suggested that about 15% of comprehension of metaphorical sentences can be regarded as resulting from a superposition state of literal and metaphorical understanding. This result sheds new light on the emergent function and cognitive state surrounding metaphor comprehension.

## 1. Compositional and non-compositional ways of comprehending metaphorical sentences

Metaphors are a bases for creating and understanding novel concepts (Holyoak and Stamenković, 2018). Several studies have pointed to the features of emergence in metaphor comprehension, arising from the interaction between the target and source (Cameron and Deignan, 2006). Furthermore, in metaphorical sentences, literal and figurative understanding can coexist. Does this coexistence create any novel meaning?

Consider the sentence, “Bob is a baby,” which has at least two possible meanings. One is the literal meaning, “Bob is actually an extremely young child.” The other is the figurative meaning, “Bob is not a mature person.” Even if a sender intended to convey the figurative meaning and the receiver could understand the sender's intention based on the context, the receiver could simultaneously imagine an actual baby while reading or listening to this sentence, and this image may affect their comprehension of the sentence. In this line of thinking, we assume the meaning of a metaphorical sentence comprises the coexisting literal and figurative meanings.

Previous studies have focused on a different angle, namely the differences in the process of literal and figurative understanding (Keysar, 1989; Giora, 2002), and found that the timing and cognitive load of each process seem to be equal. The salience, familiarity, or aptness of literal/figurative meanings are more plausible contenders to affect the process of metaphor comprehension (Holyoak and Stamenković, 2018). However, these studies implicitly assume that people choose only one meaning, either literal or figurative, and have given little consideration to possible coexistence and its impact on the comprehension.

**Figure 1:**
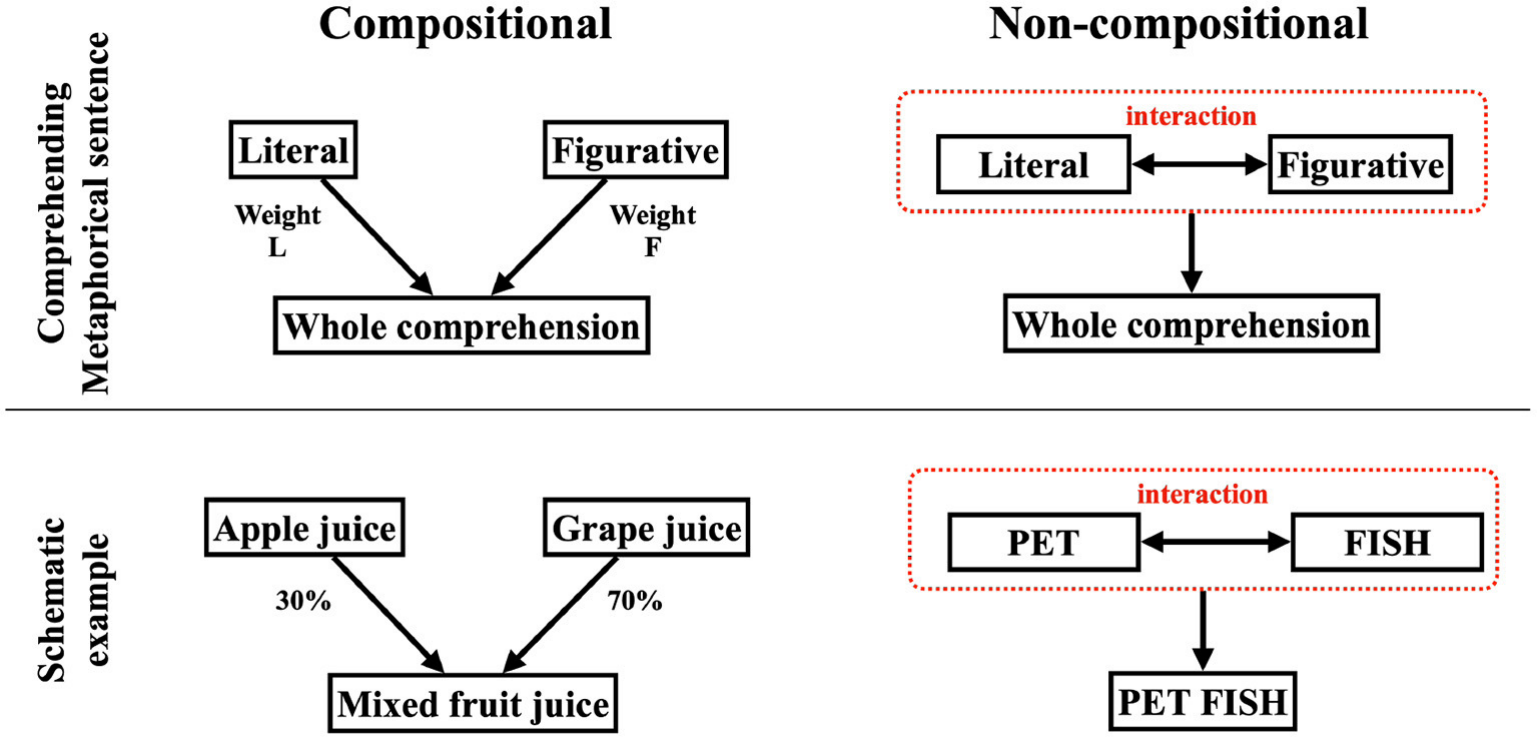
Explanation of compositional and non-compositional. The **(right)** parts show the non-compositional case and the **(left)** parts show the compositional case.

This article aims to deepen our understanding of metaphor comprehension by examining how a comprehensive understanding of metaphorical sentences is represented. Assuming the whole meaning of a metaphorical sentence is constructed based on the literal and figurative meanings, there are two possible ways they could integrate (Figure 1).

One way is composing the literal and metaphorical meanings. The whole meaning can be reduced to a kind of weighted sum of the literal and figurative (Figure 1, left illustrates this case). This model includes the case where the whole meaning is represented by either the literal or figurative meaning alone. Models that represent the meaning of words as a vector in a high-dimensional semantic space, such as word2vec (Mikolov et al., 2013) or Latent Semantic Analysis, usually model sentences as a sum of each word's vector. One schematic example is a mixed fruit juice made from mixing 30% apple juice and 70% grape juice. This mixed juice is a composition of apple and grape juice, nothing more and nothing less. In cognitive science, BLACK CAT is one of the compositional examples in the study of conceptual combination (Bruza et al., 2015).

Another way is non-compositional, where a novel meaning is created that is non-reducible to literal and metaphorical (Figure 1, right illustrates this case). The non-compositional route can be considered a kind of emergent. To understand non-compositionality, the guppy effect is helpful. Although we feel GUPPY is neither a prototypical PET or prototypical FISH, GUPPY is a prototypical PET FISH (Gabora and Aerts, 2002; Aerts, 2009; Wang et al., 2014). This intuition suggests that the meaning of “PET FISH” cannot be reduced to “PET” and “FISH,” and this understanding is an example of non-compositionality.

We assume non-compositionality as one possible function of a metaphorical sentence. For instance, suppose we wish to send either a literal or figurative message. In that case, we can just use a literal meaning sentence (Bob is actually an extremely young child) or a figurative meaning sentence (Bob is not a mature person) with less ambiguity, instead of the metaphorical sentence.

While various non-compositional schemes are possible, this article hypothesizes that the superposition state between literal and figurative meaning is one possible model of non-compositional comprehension. The superposition state, which is based on the quantum probability theory (also called the non-commutative probability theory) and employed mainly in quantum physics, can model the interaction between multiple states to create a novel state.

In recent years, cognitive science and psychology have also employed the quantum probability theory to model cognition (Gabora and Aerts, 2002; Aerts, 2009; Busemeyer and Bruza, 2012; Yearsley, 2017), and for a review (Pothos and Busemeyer, 2022). This field, termed “quantum cognition,” comprises several studies in decision-making, conceptual combination, perception, memory, and more. With regard to this article, Gabora and Aerts (2002) argued that previous theories could not explain the emergence or loss of features arising from the combination of concepts and proposed a context-aware model using *SCOP* (state context property) formalism, which is based on quantum mechanics. They utilized their model to explain the guppy effect by calculating context-dependent distances between concepts. Gabora and Kitto (2017) also applied SCOP to propose a quantum theory of humor. They modeled the interpretation state of jokes as a superposition of multiple possible interpretations of the sentences or words explaining the funniness of jokes (The superposition state is further explained in Section 2). Many other models using superposition states have been proposed; for example, Aerts (2009) proposed a model of conceptual combination using superposition states, and Bruza and Woods (2008) modeled the meaning of a word as a superposition state of multiple potential meanings, the collapse of which would settle on a single meaning. Surov et al. (2021) used the superposition state to model text perception and entangled states to represent the two-concept perception. They also discussed the possible neural basis for quantum cognitive modeling. Bruza et al. (2015) applied the entangled states to model non-compositional conceptual combinations and verified the non-compositionality using Bell type inequalities.

This article proposes an original model in which metaphor comprehension is represented as a superposition state, and empirically tests the hypothesis using a simple method, modified for our purpose. In Section 2, we explain the idea of modeling non-compositional comprehension of metaphorical sentences using a superposition state and share a brief explanation of quantum cognition. Subsequently, we describe the method used to test our model, involving previous experiments of order effect and *QQ equality* (Wang et al., 2014) in Section 3. Section 4 explains the experimental setup, and Section 5 shares the results of the experiments and model comparison. Finally, we discuss the possibility of applying a superposition state theory to elucidate the process of metaphor comprehension (Section 6). In this last section, we also point out the implication of the superposition state as metaphor comprehension.

## 2. Modeling non-compositionality as a superposition state based on quantum cognition

To explain the superposition state concerning non-compositionality, we share the famous double-slit experiment in physics.

**Figure 2:**
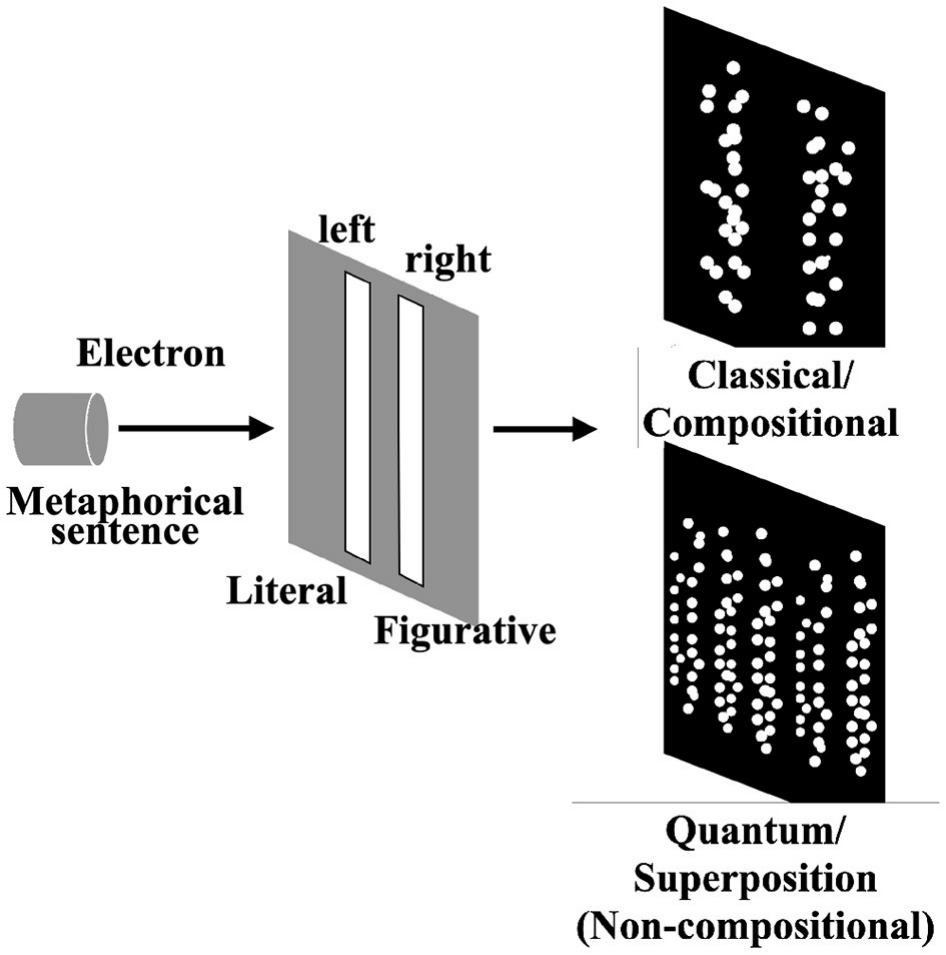
Double slit experiment and schematic explanation of superposition states. The **(upper)** parts show the classical case but the result of the double slit experiment follows the **(lower)** case with interference fringe.

In the experiment, an electron gun fires electrons into a photographic dry plate one by one. Between the electron gun and plate, another plate with a double-slit is placed. The electrons can reach the photographic dry plate only if they pass through either slit. Suppose the electron is a particle like a ball, that follows classical physics based on classical probabilities; the distribution of particles observed on the dry plate would then correspond to the sum of the two bell-shaped distributions, with two peaks just below the slits (Figure 2, upper). However, the result of this experiment showed a distribution with fringe-like waves of interference (Figure 2, lower). This result follows the prediction of the quantum physics built on the quantum probability theory, due to the quantum nature of the electron. In this experiment, the state of each electron is a superposition of the state of passing through the right slit and through the left slit, which explains the interference fringe pattern^1^.

Analogous to the double-slit experiment, we explain our idea of comprehending metaphorical sentences (Figure 2). Imagine we read a metaphorical sentence that can be understood in both literal and figurative ways. If we interpret the sentence in only one way (literal/figurative) at a time, the situation corresponds to the classical case, such as firing a ball at the double slit. On the contrary, if our overall interpretation cannot be reduced to the composition of each interpretation, it can be regarded as a superposition state, and the result of the measurement follows the quantum case and shows the “interference fringe.”

Mathematically, we modeled our idea as follows. At first, as in previous studies on quantum cognition (Aerts, 2009; Busemeyer and Bruza, 2012; Gabora and Kitto, 2017; Surov et al., 2021; Pothos and Busemeyer, 2022), we represent the cognitive state as a state vector ψ. The state of having a literal understanding is represented as ψ_*l*_, and figurative understanding is represented as ψ_*f*_.

When the states of comprehension are in the superposition state, we represent the states of the readers' comprehensive comprehension of the metaphorical sentence as ψ_*whole*_.


(1) $$\begin{array}{l} \psi_{whole}=c_{1}\psi_{l}+c_{2}\psi_{f}\;c_{1},c_{2}\in C,c_{1}^{2}+c_{2}^{2}=1 \end{array}$$

Note that the *c*_1_ and the *c*_2_ are probability amplitudes, not probability itself.

When we measure the states of comprehension, the probability distributions are represented as in Equation (2).


(2) $$\begin{array}{l} \begin{array}{l} \langle \psi_{whole}|P\left(a\right)|\psi_{whole}\rangle =|c_{1}{|}^{2}\langle \psi_{l}|P\left(a\right)|\psi_{l}\rangle +|c_{2}{|}^{2}\langle \psi_{f}|P\left(a\right)|\psi_{f}\rangle \\ \;+c_{1}^{*}c_{2}\langle \psi_{l}|P\left(a\right)|\psi_{f}\rangle +c_{2}^{*}c_{1}\langle \psi_{f}|P\left(a\right)|\psi_{l}\rangle \end{array} \end{array}$$

We use bracket notation where 〈*x*| represents a row vector and |*x*〉 represents a column vector in Hilbert space. *P*(*a*) is the projection operator on vector *a*. In this case, 〈ψ_*whole*_|*P*(*a*)|ψ_*whole*_〉 denotes the probability that the reader whose state is ψ_*whole*_ agrees with the interpretation “a” for the sentence. The 〈ψ_*l*_|*P*(*a*)|ψ_*l*_〉 indicates the probability that the reader whose state is ψ_*l*_ agrees with the interpretation “a” for the sentence. The 〈ψ_*f*_|*P*(*a*)|ψ_*f*_〉 indicates the probability that the reader whose state is ψ_*f*_ agrees with the interpretation “a” for the sentence.

The last two terms of Equation (2) are together called the “interference term.” This term characterizes the superposition state, which cannot be reduced to the composition of each contributing state.

Conversely, when the states of comprehension follow the classical probability theory, the addition of multiple states can only be represented as mixed states [for an explanation using density matrices, see Yearsley (2017)]. Then the probability distribution is reduced to the following Equation 3.


(3) $$\begin{array}{l} \begin{array}{c} \langle \psi_{whole}|P\left(a\right)|\psi_{whole}\rangle =|c_{1}{|}^{2}\langle \psi_{l}|P\left(a\right)|\psi_{l}\rangle +|c_{2}{|}^{2}\langle \psi_{f}|P\left(a\right)|\psi_{f}\rangle \end{array} \end{array}$$

Equation (3) denotes the probability distribution for the state in which the whole comprehension is represented as a weighted sum of the probability distributions for the states of literal and figurative comprehension. Therefore, we can regard this as the compositional case.

In sum, based on our model, we can distinguish between a non-compositional case, corresponding to a superposition state, and a compositional case by the presence or absence of an interference term.

As mentioned in Section 1, various studies have been done on concept combination and text comprehension using the superposition state. Based on these studies' findings, this study is the first investigations into metaphor comprehension using the framework of quantum cognition from the perspective of literal and figurative meaning compositionally. Regarding the empirical method, we applied QQ equality to test our model (see Section 3). Previous studies have mainly tested for the existence of quantum effects, either by checking the violation of the law of total probability or Bell's inequality (Busemeyer and Bruza, 2012; Gabora and Kitto, 2017), or by comparing quantum and classical models directly (Busemeyer et al., 2019; Kvam et al., 2021). However, the verification of Bell's inequality to test compositionality involves psychology-specific control difficulties (Bruza et al., 2015), and the direct comparison requires the construction of a quantum-type model and an alternative model, both of which can be evaluated by data. To alleviate these difficulties, this article proposes the application of QQ equality as a simple method and a first step toward the verification of quantum effects to test the compositionality.

It must be noted that our model does not consider the influence of context, such as each participant's prior knowledge and affective state, on the interpretation state. The model can be regarded as dealing with the sum of all possible contextual states. This point is discussed as a limitation and avenue for future research in Section 6. In addition, metaphorical sentences can be interpreted in more than two ways, depending on the context. In this study, as a first step, we focused our examination on the two most conventional interpretations (typical literal and figurative meanings) to simplify the model (similar to the approach of Gabora and Kitto, 2017) and apply the QQ equality.

## 3. Testing the hypothesis using the QQ equality

We employed QQ equality to test our hypothesis. Wang et al. (2014) proposed QQ equality as the quantitative prediction regarding order effect based on the framework of quantum cognition. With the notation *P*(*A*_*yes*_ &* then B*_*no*_) indicating the probability of a pair of answers, wherein the answer to question A is yes, and then B is no, QQ equality is represented as Wang et al. (2014) and Pothos and Busemeyer (2022):


(4) $$\begin{array}{l} q\;value=\left[P\left(A_{yes}\;\&\;then\;B_{no}\right)+P\left(A_{no}\;\&\;then\;B_{yes}\right)\right]\\ \;-\left[P\left(B_{yes}\;\&\;then\;A_{no}\right)+P\left(B_{no}\;\&\;then\;A_{yes}\right)\right]\\ \;=\left[P\left(A_{yes}\;\&\;then\;B_{yes}\right)+P\left(A_{no}\;\&\;then\;B_{no}\right)\right]\\ \;-\left[P\left(B_{yes}\;\&\;then\;A_{yes}\right)+P\left(B_{no}\;\&\;then\;A_{no}\right)\right]\\ \;=0 \end{array}$$

Wang et al. (2014) reported on the consistency of predictions from QQ equality and the results of 70 national surveys in the USA, concluding that their model was indeed supported.

QQ equality is not a specific relation for the order effect, but more a general relation for the observable states derived from the interference term of the superposition states (see the supporting information of Wang et al., 2014). Thus, if a cognitive state can be described by a superposition state, by changing the order of observations, we can find the relation suggested by QQ equality in the experimental result.

Based on this notion, we applied the QQ equation to test our model where the comprehensive comprehension of a metaphorical sentence is modeled as the superposition states of the literal and figurative understanding. When we measure the literal and figurative understanding one after another, if our model is better than the compositional one, QQ equality will provide a better explanation of the experimental result.

In our experiment (see also Table 1), we used the notation θ_*yn*_ to represent the probability of the answer being yes to the question, “does the participant agree with the literal interpretation?” and then yes to the question, “does the participant disagree with the figurative interpretation?” θ_*yy*_, θ_*ny*_, θ_*nn*_ are similarly defined. γ_*yn*_ represents the probability of the answer yes to the question, “does the participant agree with the figurative interpretation?” and then yes to the question, “does the participant disagree with the literal interpretation?” γ_*yy*_, γ_*ny*_, γ_*nn*_ are defined similarly. *q*_*yy*_ is defined as the difference of γ_*yy*_ from θ_*yy*_, and *q*_*yn*_, *q*_*ny*_, *q*_*nn*_ are defined similarly (On Table 1, the right matrix is obtained by subtracting the center matrix from the left matrix). Based on this notation, *q value* is represented as follows.


(5) $$\begin{array}{l} \begin{array}{l} q\;value=q_{yy}+q_{nn}\\ \;=q_{yn}+q_{ny} \end{array} \end{array}$$

**Table 1:** The explanation of the *q*-value in our experiment.

**Literal-figurative**			**Figurative-literal**			**Terms of the QQ equation**		
	*L*_*y*_	*L*_*n*_		*L*_*y*_	*L*_*n*_		*L*_*y*_	*L*_*n*_
*F*_*y*_	θ_*yy*_	θ_*ny*_	*F*_*y*_	γ_*yy*_	γ_*ny*_	*F*_*y*_	*q*_*yy*_	*q*_*ny*_
*F*_*n*_	θ_*yn*_	θ_*nn*_	*F*_*n*_	γ_*yn*_	γ_*nn*_	*F*_*n*_	*q*_*yn*_	*q*_*nn*_

The left matrix represents the probabilities of the answers in the Literal first condition. The center matrix represents the probabilities of the answers in the Figurative first condition. The right matrix is obtained by subtracting the center matrix from the left matrix, represented in the QQ equation's terms.

QQ equation means that the expected q value becomes zero. Thereafter, by checking QQ equation, we can test our model, which hypothesizes that the comprehension of metaphorical sentences involves the superposition of the literal and figurative meanings.

## 4. Experiment

We employed 21 metaphorical sentences that could be interpreted as both figurative and literal. Participants read a metaphorical sentence and its literal/figurative interpretation sentences, and answered whether their interpretation was consistent with this literal/figurative interpretation or not. To test our model, we analyzed whether the result fit the prediction of QQ equality or not using the model comparison technique.

### 4.1. Participants

We collected data from 1,000 participants. It was difficult to estimate the effect size in our experiment due to the absence of similar studies; we, therefore, determined the number of participants in accordance with Wang et al. (2014), which tested the QQ equality with order effect. Participants were recruited using CrowdWorks, one of Japan's most popular crowdsourcing services. The mother tongue of all the recruited participants was Japanese. According to their self-report, their ages range from 10s to 70s. Five participants were in their teens, 135 participants in their 20s, 338 participants in their 30s, 311 participants in their 40s, 155 participants in their 50s, 47 participants in their 60s, and seven participants were over 70. Only participants who answered the Instructional Manipulation Check (IMC) correctly could proceed to the main task. The participants were paid 286 yen (about two dollars), only if they finished the main task.

### 4.2. Material

We used 21 Japanese metaphorical sentences as experimental material. Every sentence could be interpreted as having both literal and figurative meaning. We adopted two metaphorical sentences from Keysar (1989) and wrote 19 metaphorical sentences according to Lakoff and Johnson (1980) and Kojiro (2011). For each metaphorical sentence, we also made a literal and figurative meaning sentence. For example, one sentence was “Kei is reaching new heights” (Original Japanese version is “ケイは登り坂にいる”). This sentence could be interpreted as both “Kei is climbing up a hill” (ケイは坂道を登っている) as a literal meaning, and “Kei is in a state where things are going well” (ケイは物事がうまくいく状態にある) as a figurative meaning. The English translations of each of the sentences are listed in Table 2.

**Table 2:** Translated metaphorical sentences and the results of the χ^2^ test.

**Sentence**	**Figurative interpretation**	**Order effect**	**QQ equality**	**Super- position**
Steven is a soldier.	Steven is someone who is at the forefront of a very competitive field.	0.063	0.044^*^	N
Augustus is a slave.	Augustus has lost his independence and is constrained in his behavior since he has lost his mind to certain things and people.	0.0017^**^	0.059	Y
Benny is a ghost.	Benny is a gloomy person with no energy.	0.0022^**^	0.034^*^	N
Jun is a raccoon.	Although Jun is seemingly innocent, he is scheming.	0.434	0.286	N
Yumi got stuck in the mud.	Yumi is in a bad situation that is difficult to resolve.	0.388	0.823	N
Kei is on an uphill.	Kei is in a state where things are going well.	0.303	0.618	N
Mike is in the maze.	Mike is in a problem that is difficult to solve.	0.940	0.934	N
Jack is at a crossroads.	Jack is in a situation where he has to make a big decision.	NaN	NaN	NaN
Yuuki is at a dead end.	Yuuki is at a dead end, unable to find a solution to his problem.	0.103	0.129	N
Today was a stormy day.	Today was a busy day with many incidents.	0.100	0.138	N
Rick is a wizard.	Rick solves problems using clever methods and novel techniques.	0.312	0.352	N
Leonard has a stomach ache.	Leonard has a difficult problem.	NaN	NaN	NaN
Rika chose a different path tp Zack.	Rika and Zack didn't share their lives and made separate choices.	0.189	0.152	N
Sunny is stepping.	Sunny is stagnating, unable to make good progress on what needs to be done.	0.050^*^	0.117	Y
Zacks is stuck.	Zacks does not know how to solve the problem and is unable to do anything about it.	NaN	NaN	NaN
Vinny crossed the pass.	Vinny got through a difficult phase.	0.180	0.822	N
Noah caught a big haul.	Noah got the opportunity to acquire large sums of money or great achievements.	NaN	NaN	NaN
Kaoru feels a tailwind.	Kaoru feels that a boosting event has taken place, giving him an advantage.	NaN	NaN	NaN
Nick fell.	Nick has fallen out of favor.	0.050	0.123	Y
Yuki is on fire.	Yuki is in a heightened state of feeling, such as motivated or angry.	0.645	0.781	N
Mika is carrying a heavy load.	Mika has burdensome things and problems.	0.161	0.854	N

The literal interpretation of the sentence is omitted since it is apparent. The column of the “order effect” and “QQ equality” represent the *p*−*value* of the χ^2^ test for them. The column of “superposition” represents if both the order effect and QQ equality is supported for the sentence. Y means Yes, N means No, and NaN means that it could not be calculated. ^*^p < 0.05.^**^p < 0.01.

### 4.3. Procedure

Each participant registered on CrowdWorks, and participated in the experiment by accessing Qualtrics online through a web browser. They were allowed to access Qualtrics using a personal computer, but not a smartphone, which was defined as the operating system being iOS or Android in the metainformation of the web browser.

After agreeing to participate in the experiment, each participant completed the IMC by which we checked that they had read the task instructions properly. Only those who passed the IMC were allowed to proceed to the main task and complete the experiment. The participants who passed the IMC proceeded to the practice exercise to ensure their understanding of the main task. The procedure and instructions for the practice exercise was the same as the main task but involved answering for just one metaphorical sentence. After the practice exercise, the participant moved on to the main task.

In the main task, the participant first read the instructions:

First, you read a sentence. Once you have read and understood the presented sentence, click the button “please click here after reading the sentence.” Then move on to the next page. On the next page, one of the possible interpretations of the sentence is randomly presented. Please answer whether the suggested interpretation is consistent with your interpretation of the sentence at the first reading during this experiment. This trial is repeated several times. Note that the presented sentences can be interpreted in multiple ways. Therefore, you can answer “consistent” to more than one interpretation (irrespective of how many times you have previously answered “consistent” or “inconsistent”). There is no correct answer. Please answer according to your own interpretation.

**Figure 3:**
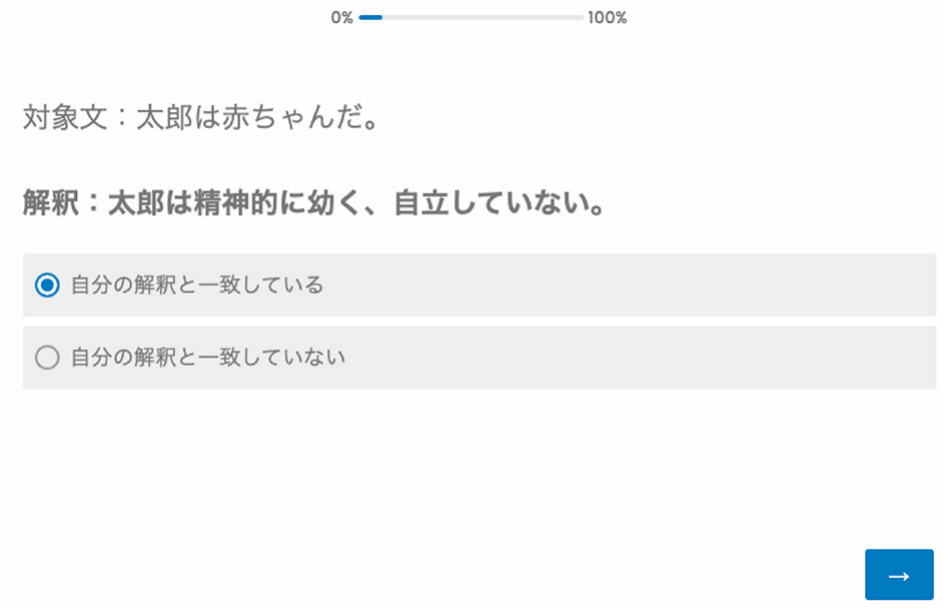
Screen capture of the task. The top sentence is the target metaphorical sentence. The second sentence is the interpretation sentence. The third sentence translates to “Consistent with my interpretation.” The fourth sentence translates to “Inconsistent with my interpretation.” Participants chose either the third or fourth sentence, then clicked the blue button on the bottom right to move on to the next page.

After reading these instructions, the participant moved on to the next page. Subsequently, they read one metaphorical sentence and clicked the button with the instruction “Please click here after reading the sentence” to move to the next page. After clicking, a literal or figurative interpretation of the metaphorical sentence was presented to the participant. The participant clicked “Consistent with my interpretation” or “Inconsistent with my interpretation.” The participant could see the target metaphorical sentence on the same screen (see Figure 3). After answering and moving on to the next page, the other interpretation (figurative or literal) of the metaphorical sentence was presented. Again, the participant clicked “Consistent with my interpretation” or “Inconsistent with my interpretation,” and moved on to the next page. The order of presentation of literal and figurative interpretations was randomized. After responding to both the literal and figurative interpretations, a new metaphorical sentence was presented. The order of presentation of the metaphorical sentence was also randomized. When all the 21 metaphorical and 42 interpretation sentences were presented to the participant, the experiment was considered completed.

## 5. Results

### 5.1. The relation of QQ equality

Since one participant did not report the completion of the task on CrowdWorks, we excluded their data from the analysis. We also excluded data from participants whose response times were in the top or bottom 5%. As a result, the number of valid responses analyzed was 949.

The analysis basically followed the steps described in Wang et al. (2014) to test whether the participants' answers were in alignment with the QQ equality.

At first, Wang et al. (2014) selected the larger order effect to analyze. In our case, for each metaphorical sentence, we compared |*q*_*yy*_+*q*_*nn*_| and |*q*_*yn*_+*q*_*ny*_| (we refer to these terms as “the size of the order effect” below) and chose the corresponding pair of terms to analyze. If |*q*_*yy*_+*q*_*nn*_| was larger, we chose to analyze the diagonal q value, *q*_*yy*_−*q*_*nn*_. Otherwise, we chose to analyze the anti-diagonal q value, *q*_*yn*_−*q*_*ny*_.

**Figure 4:**
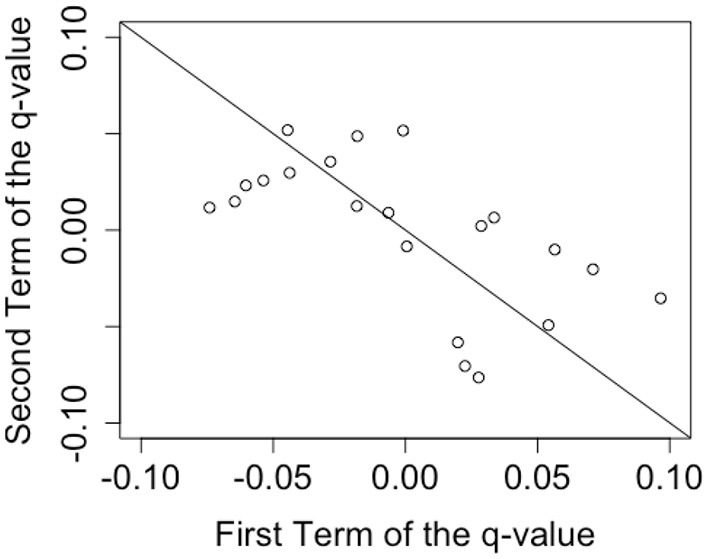
Scatter plot of the q value terms. The prediction of the QQ equality suggests that the data points fall on the line with the intercept of zero and the slope of -1 shown on the figure.

**Figure 5:**
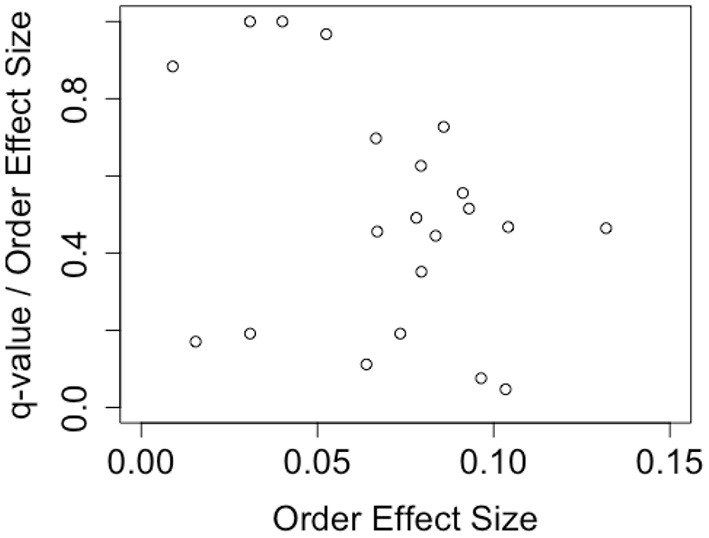
Normalized q values plotted as a function of the size of the order effect.

To confirm the distribution of the data, the scatter plot of the pairs of *q*_*yy*_ and *q*_*nn*_, or *q*_*yn*_ and *q*_*ny*_, which were selected in the previous step (Figure 4) are shown. If the q value was equal to zero, the plot should fall along a line with the intercept of zero and slope of −1 (the line in Figure 4). The data seems to follow this straight line, similar to the result of Wang et al. (2014). We also plotted the ratio of (*qvalue*)/(*sizeofordereffect*) on the right side of Figure 5. Since the size of the q value is bounded by the size of the order effect, Wang et al. (2014) normalized the q value by the size of the order effect and plotted it. Figure 5 shows that some of our q values remained small when normalized by the size of the order effect, but others did not. This result suggested that some of the comprehensions of the metaphorical sentences in this study could be regarded as being the result of a superposition state, but others could not.

Next, we conducted a model comparison using the χ^2^ test to assess the order effect and QQ equality. Although, Figures 4, 5 illustrate the data distribution, a statistical test was needed for additional confirmation. Based on Wang et al. (2014), we conducted two tests corresponding to the order effect and QQ equality. Support for both order effect and QQ equality would indicate the existence of the superposition states.

The method of testing order effect followed Wang et al. (2014), in which they compared the model restricted by the relation of order effect with the null model using the χ^2^ test. To test QQ equality for our data, we improved the model of QQ equality of Wang et al. (2014). The model of Wang et al. (2014) was too weak to represent QQ equality. Their model only represented significant relations between the terms of q value (in other words, q value is restricted to a constant), and did not represent q value equal to zero. We obtained the constraints of the QQ equation as a problem of constrained multinomial distribution using the Lagrange undetermined multiplier method. The model with these constraints was employed as the model of QQ equality and compared with the null model (see Supplementary material for detail on our model).

Similar to Wang et al. (2014), based on the model comparison method using χ^2^ test, when the *p*−*value* for the test of order effect was significant, the order effect was significantly suggested, since the model that allows order effects is less constrained than the model of no order effect. When the *p*−*value* for the test of QQ equality was not significant, the QQ equality was suggested because the model restricted by QQ equality is more constrained than the null model.

Every participant disagreed with the same interpretations for five sentences on the same condition. Therefore, some of the θ_*yy*_ or γ_*nn*_ were zero, and we excluded these five sentences from the analysis since we could not calculate the values of χ^2^ (in Table 2, NaN corresponded to this exclusion). Finally, we analyzed 16 sentences.

Table 2 shows the results of the χ^2^ test for the order effect and QQ equality. Among the 16 sentences, at the 5% significance level, the order effect was significant in three sentences, and QQ equality was supported in 14 sentences. In sum, three of the 16 sentences were regarded to be in a superposition state.

## 6. Discussion

### 6.1. Summary, limitation, and the future work

This article hypothesizes that the comprehension of a metaphorical sentence involves the non-compositional state of literal and figurative understanding, and can be modeled as their superposition state. We also proposed a novel method to test this hypothesis. The results suggested that three of the 16 metaphorical sentences' comprehensions could be regarded as involving a superposition state.

This result suggested that comprehension of about one fifth of the metaphorical sentences can be regarded as superposition, and therefore, non-compositional. Metaphorical sentences have many characteristics, such as familiarity, aptness, salience, conventionality, and interpretive diversity (Giora, 2002; Utsumi, 2007; Holyoak and Stamenković, 2018). Currently, due to the small number of sentences employed in this experiment, it is unclear whether the sentences which can be comprehended as superposition states have any common features, and if so, what characteristics they share. Further study is required to clarify this point.

We should also point out that the significance of the order effect mainly restricted the result. QQ equality was originally proposed to test the order effect (Wang et al., 2014), but the size of the order effect of the understanding of metaphorical sentence was small or not significant. Using QQ equality is a simpler method to test the presence of quantum effects than modeling superposition states directly. Hence, as the first step to test the existence of superposition, QQ equality is a reasonable method. However, because of this limitation, combining other methods is needed to obtain more detailed and robust results.

The context of each participant, such as their prior knowledge or affective state, could also affect the results. The model we proposed in this article did not employ “context” as a variable, and we also did not control the context of the participants. Thus, this study can be regarded as implicitly assuming the average state of the participants' interpretive state before reading the metaphorical sentences in the experiment to be a context-independent state. Although this assumption has been implicitly employed in some studies (Wang et al., 2014; Bruza et al., 2015; Kvam et al., 2021), others have pointed out the significant influence of context on the initial condition of participants (Gabora and Aerts, 2002; Gabora and Kitto, 2017).

Considering the diversity of the participants' initial states involved in interpreting metaphorical sentences, providing contextual information to participants could improve the study. For example, for the target sentence “Today was a stormy day,” we can provide the sentence “I hear the sound of heavy rain” or “Many unexpected events occurred” as a context before reading the target sentence. The first contextual sentence would bring the participants' interpretive states closer to the literal meaning, while the second would bring them closer to the figurative meaning. When only one contextual sentence is given, the superposition state disappears (collapses), and neither the order effect nor QQ equality is significant, since only one interpretation becomes dominant. When both contextual sentences or a more ambiguous contextual sentence such as “I felt soaking wet” is given, the participants' states would be closer to the specific superposition state of the literal and figurative meanings. In conducting experiments with initial conditions based on this approach, clearer results can be expected.

### 6.2. Reinterpretation of superposition states and suggestions from the results

**Figure 6:**
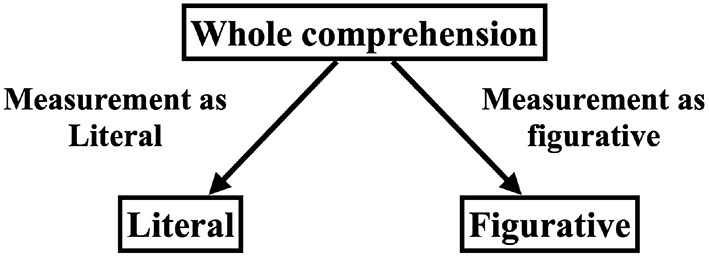
Comprehensive first interpretation.

Despite these limitations, by revisiting the superposition states, the results of this study indicate one more perspective of comprehension of metaphorical sentences. Given the consideration of the double-slit experiment, the superposition of metaphorical sentences can be interpreted in two ways. One way is the interpretation described in Section 1 and Figure 1. The comprehension of the metaphorical sentence emerges from the literal and figurative understanding. According to this interpretation, the literal and figurative understandings come first, and then the comprehensive comprehension emerges. Another interpretation, which is closer to that in the quantum physics field such as that of the double slit experiment, is that comprehensive comprehension comes first, following which, we evaluate or categorize the sentence as a literal or figurative understanding (Figure 6). We call this the “comprehensive first interpretation.”

Based on the comprehensive first interpretation, the original state of the whole comprehension cannot be seen or known. By selecting the way of measurement or the type of interpretation, we may get results accordingly. In the double-slit experiment, the interference fringe indicates the electron passing through both slits simultaneously, but when we measured its position on the dry plate, it is fixed at a single point. In our experiment, the interference fringe corresponded to the interference term, which was measured as QQ equality, and the position correspond to the way of interpretation. Analogically, once we measure the literal/figurative interpretation, the interpretation becomes fixed, and we get the answer. Furthermore, comprehension is not restricted to literal or figurative. If we can select another type of interpretation, we can understand the sentence in a new way.

Previous studies assumed that a sentence has a discrete meaning, and in the case of the metaphorical sentence, there are two possible discrete meanings: literal and figurative. Based on this assumption, whether compositional or non-compositional, readers reach the literal or figurative understanding first. On the contrary, based on the comprehensive first interpretation, readers initially reach an amalgamated meaning by which we can access various types of understanding based on our interpretations which may be restricted by our knowledge or culture.

Comprehensive first interpretation is still a possible theoretical hypothesis and should be tested empirically. We expect that ongoing study on quantum cognition would be valuable to the understanding of metaphor comprehension from various perspectives.

## Data availability statement

The raw data supporting the conclusions of this article will be made available by the authors, without undue reservation.

## Ethics statement

The studies involving human participants were reviewed and approved by Ethics Review Committee on Research with Human Subjects of Waseda University. The patients/participants provided their written informed consent to participate in this study.

## Author contributions

MF was responsible for conducting an experiment and data analysis, manuscript has been written, and contributed to the article and approved the submitted version.

## Footnotes

^1^It is difficult to imagine the state where one electron passes through both slits simultaneously, like a wave, and is measured as one particle on the dry plate in the distribution with the interference fringe. However, quantum physics explains these phenomena precisely. This result shows the limitation in our imagining of nature. The limitation in our image of cognition may be suggested by experiments examining phenomena that follow the quantum probability theory.
