# Supplementary material for: Does the coexistence of literal and figurative meanings in metaphor comprehension yield novel meaning?: Empirical testing based on quantum cognition
Source: Front Psychol. 2023 Mar 30;14:1146262. doi: 10.3389/fpsyg.2023.1146262 (PMC10098108; doi:10.3389/fpsyg.2023.1146262)
Supplement: Supplementary file 1 [file Presentation_1.pdf]

Supplementary Material

1 PARAMETER ESTIMATION FOR THE MODEL FOLLOWING QQ EQUALITY

Wang et al. (2014) estimate the log-likelihood for the model constrained by the QQ equality in their supporting information. However their model corresponding to the estimated log-likelihood indicates that there are some relationships between q_{yy} (q_{yn}) and q_{nn} (q_{ny}) and do not represent $q_{yy} - q_{nn} = 0$ or $q_{yn} - q_{ny} = 0$. Therefore, their model is weaker than the one indicated by QQ equality.

We improved on this point and estimated the log-likelihood for the model constrained by the QQ equality. We performed the maximum likelihood estimation for the probability of each response constrained by QQ equality using Lagrange's method of undetermined multipliers.

Consider f_{yn} as the frequency of the answer yes to the question, "does the participant agree with the literal interpretation?", and then yes to the question, "does the participant disagree with the figurative interpretation?" f_{yy}, f_{ny}, f_{yn} are defined similarly. Consider l_{yn} as the frequency of the answer yes to the question, "does the participant agree with the figurative interpretation?", and then yes to the question, "does the participant disagree with the literal interpretation?" l_{yy}, l_{ny}, l_{yn} are defined similarly. The notation of the probability of each case is same in the section 3 of the main manuscript.

In this case, f_{yy},f_{yn},f_{ny}, and f_{nn} follow a multinominal distribution with parameters θ_{yy}, θ_{yn}, θ_{ny}, and θ_{nn} respectively. Similarly, l_{yy},l_{yn},l_{ny}, and l_{nn} follow a multinominal distribution with parameters γ_{yy}, γ_{yn}, γ_{ny}, and γ_{nn} respectively.

The parameters constraints are as follows with a notation $I = \{yy, yn, ny, nn\}$:

$$\sum_{i=I} \theta_i = 1, \sum_{i=I} \gamma_i = 1$$
$$\forall i \in I, \theta_i, \gamma_i \geq 0 \tag{A.1}$$

The probability function of this multinominal distributions can be written as follows with $n_f = \sum_{i \in I} f_i$ and $n_l = \sum_{i \in I} l_i$:

$$g(\theta_{i\in I}|n_f, \theta_{i\in I}) = \frac{n_f!}{f_{yy}!f_{yn}!f_{ny}!f_{nn}!}\theta_{yy}^{f_{yy}}\theta_{yn}^{f_{yn}}\theta_{ny}^{f_{ny}}\theta_{nn}^{f_{nn}} \tag{A.2}$$
$$g(\gamma_{i\in I}|n_l, \gamma_{i\in I}) = \frac{n_l!}{l_{yy}!l_{yn}!l_{ny}!l_{nn}!}\gamma_{yy}^{l_{yy}}\gamma_{yn}^{l_{yn}}\gamma_{ny}^{l_{ny}}\gamma_{nn}^{l_{nn}} \tag{A.3}$$

The constrain of the QQ equality is as follow:

$$\theta_{yy} - \gamma_{yy} = -(\theta_{nn} - \gamma_{nn}) \tag{A.4}$$

This constrain equivalent of $\theta_{yn} - \gamma_{yn} = -(\theta_{ny} - \gamma_{ny})$.

Then we used Lagrange's method of undetermined multipliers. The Lagrangian function under this condition, ignoring the constant term, is as follows. Note that $\lambda_1, \lambda_2, \lambda_3$ are constant.:

$$H(\theta_i \in I, \gamma_i \in I) = \sum_{i\in I}(f_i \log\theta_i + l_i \log\gamma_i) - \lambda_1(\sum_{i\in I}\theta_i - 1) - \lambda_2(\sum_{i\in I}\gamma_i - 1) - \lambda_3(\theta_{yy} + \theta_{nn} - \gamma_{yy} - \gamma_{nn}) \tag{A.5}$$

We partial differentiated Equation A.5 for theta and transformed the equations. Then we obtained the following maximum likelihood estimation. Note $f_0 := f_{yy} + f_{nn}, l_0 := l_{yy} + l_{nn}$.

$$\theta_{yy} = \frac{f_{yy}(f_0 + l_0)}{f_0(n_f + n_l)} \tag{A.6}$$

$$\theta_{yn} = \frac{f_{01}(f_1 + l_1)}{f_1(n_f + n_l)} = \frac{f_{01}(n_f + n_l + f_0 - l_0)}{(n_f - f_0)(n_f + n_l)} \tag{A.7}$$

$$\theta_{ny} = \frac{f_{10}(f_1 + l_1)}{f_1(f_n + l_m)} = \frac{f_{10}(n_f + n_l + f_0 - l_0)}{(n_f - f_0)(n_f + f_l)} \tag{A.8}$$

$$\theta_{nn} = \frac{f_{11}(f_0 + l_0)}{f_0(n_f + n_l)} \tag{A.9}$$

$$\gamma_{yy} = \frac{l_{00}(f_0 + l_0)}{l_0(n_f + n_l)} \tag{A.10}$$

$$\gamma_{yn} = \frac{l_{01}(f_1 + l_1)}{l_1(n_f + n_l)} = \frac{l_{01}(n_f + n_l + f_0 - l_0)}{(n_l - l_0)(n_f + n_l)} \tag{A.11}$$

$$\gamma_{ny} = \frac{l_{10}(f_1 + l_1)}{l_1(n_f + n_l)} = \frac{l_{10}(n_f + n_l + f_0 - l_0)}{(n_l - l_0)(n_f + n_l)} \tag{A.12}$$

$$\gamma_{nn} = \frac{l_{11}(f_0 + l_0)}{l_0(n_f + n_l)} \tag{A.13}$$

We used these parameters to calculate the likelihood for the model comparison.

REFERENCES

Wang, Z., Solloway, T., Shiffrin, R. M., and Busemeyer, J. R. (2014). Context effects produced by question orders reveal quantum nature of human judgments. *Proceedings of the National Academy of Sciences* 111, 9431–9436
